# cGAS-STING effectively restricts murine norovirus infection but antagonizes the antiviral action of N-terminus of RIG-I in mouse macrophages

**DOI:** 10.1080/19490976.2021.1959839

**Published:** 2021-08-04

**Authors:** Peifa Yu, Zhijiang Miao, Yang Li, Ruchi Bansal, Maikel P. Peppelenbosch, Qiuwei Pan

**Affiliations:** a Department of Gastroenterology and Hepatology, Erasmus MC-University Medical Center, Rotterdam, The Netherlands; b Translational Liver Research, Department of Medical Cell Biophysics, Technical Medical Centre, Faculty of Science and Technology, University of Twente, Enschede, The Netherlands

**Keywords:** Murine norovirus, cGAS, STING, RIG-I, ISGs

## Abstract

Although cyclic GMP-AMP synthase (cGAS)-stimulator of interferon genes (STING) signaling has been well recognized in defending DNA viruses, the role of cGAS-STING signaling in regulating infection of RNA viruses remains largely elusive. Noroviruses, as single-stranded RNA viruses, are the main causative agents of acute viral gastroenteritis worldwide. This study comprehensively investigated the role of cGAS-STING in response to murine norovirus (MNV) infection. We found that STING agonists potently inhibited MNV replication in mouse macrophages partially requiring the JAK/STAT pathway that induced transcription of interferon (IFN)-stimulated genes (ISGs). Loss- and gain-function assays revealed that both cGAS and STING were necessary for host defense against MNV propagation. Knocking out cGAS or STING in mouse macrophages led to defects in induction of antiviral ISGs upon MNV infection. Overexpression of cGAS and STING moderately increased ISG transcription but potently inhibited MNV replication in human HEK293T cells ectopically expressing the viral receptor CD300lf. This inhibitory effect was not affected by JAK inhibitor treatment or expression of different MNV viral proteins. Interestingly, STING but not cGAS interacted with mouse RIG-I, and attenuated its N-terminus-mediated anti-MNV effects. Our results implicate an essential role for mouse cGAS and STING in regulating innate immune response and defending MNV infection. This further strengthens the evidence of cGAS-STING signaling in response to RNA virus infection.

## Introduction

Noroviruses are positive sense single-stranded RNA viruses belonging to the *Caliciviridae* family.^1^ The lack of robust cell culture system for human norovirus (HuNV) impedes development of effective antiviral therapeutics. The closely related murine norovirus (MNV) shares similar structural and genetic features with HuNV and efficiently propagates in vitro and in vivo, representing as a useful model for studying norovirus biology.^2^ The MNV genome is approximately 7.5 kilo bases in length, consisting of four open reading frames (ORFs). ORF1 encodes a polyprotein that is post-translationally cleaved into six non-structural proteins (NS1/2 to NS7), while ORF2 and ORF3 encode the major and minor structural viral proteins as VP1 and VP2, respectively. ORF4 overlaps with ORF2, and encodes the virulence factor (VF1), which has been reported to antagonize innate immune response.^3^

Innate immune response plays a key role in the early recognition and restriction of viral infection. In the cytoplasm, viral RNA is mainly sensed by retinoic acid inducible gene-I (RIG-I)-like receptors (RLRs) including RIG-I and melanoma differentiation associated gene 5 (MDA5), and Toll-like receptors (TLRs).^4,5^ Upon recognition, the RNA-stimulated signaling proceeds through adaptor mitochondrial antiviral signaling (MAVS; also called IPS-1, VISA, and Cardif) protein that activates transcription factors such as nuclear factor-ĸB (NF-ĸB) and interferon (IFN)-regulatory factors 3 (IRF3), which then translocate into the nucleus to drive secretion of various cytokines including IFNs, the potent inhibitors of viral replication.^6–9^ Cytosolic DNA derived from pathogens is recognized by a DNA binding protein, cyclic GMP-AMP (cGAMP) synthase (cGAS).^8,10^ Upon viral DNA recognition, cGAS produces 2ʹ3ʹ-cGAMP, which engages an endoplasmic reticulum (ER)-localized protein stimulator of interferon genes (STING; also called MITA, TMEM173, MPYS, and ERIS).^8,11^ Binding of 2ʹ3ʹ-cGAMP to STING induces a conformational change and activates the following transcription factor IRF3, leading to expression of IFNs.^8,12^ The released IFNs can bind to their receptors and activate Janus kinase (JAK)/signal transducer and activator of transcription (STAT) signaling pathway, initiating transcription of hundreds of IFN-stimulated genes (ISGs). A subset of ISGs are considered as the ultimate antiviral effectors limiting viral replication, including norovirus.^9,13–15^

Besides the well-established role in innate immune responses to DNA viruses, emerging evidence indicates that cGAS-STING signaling is also involved in restricting RNA virus replication.^16^ For instance, cells or mice that are deficient in cGAS or STING facilitate replication of several RNA viruses, such as vesicular stomatitis virus (VSV), Sendai virus (SeV), dengue virus (DENV), hepatitis C virus (HCV), and West Nile virus (WNV).^11,17–20^ Moreover, cGAS and STING have been reported to be associated with RNA virus-induced immune responses. Cells lacking cGAS or STING show defects in IFN activation in response to the infection of some RNA viruses, including SeV, VSV, IAV and Zika virus.^21,22^ Unlike DNA virus, RNA virus infection may not lead to quick ubiquitination and phosphorylation of STING, while SeV infection can induce STING expression.^23^ Although the association between STING with RLRs in RNA virus-mediated immune responses has been indicated, the underlying mechanism remains unclear and awaits for further study. For norovirus, MDA5 has been recognized as a sensor mediating host immune response upon MNV infection,^24^ and both MDA5 and RIG-I overexpression can restrict HuNV and MNV replication in vitro.^25–27^

To date, whether the cGAS-STING signaling plays a role in response to norovirus infection is still unknown. Thus, we investigated the potential involvement of cGAS-STING signaling mediated antiviral cellular response against MNV infection in this study.

## Results

### Regulation of STING agonists and inhibitor on MNV replication in mouse macrophages

**Figure 1.**
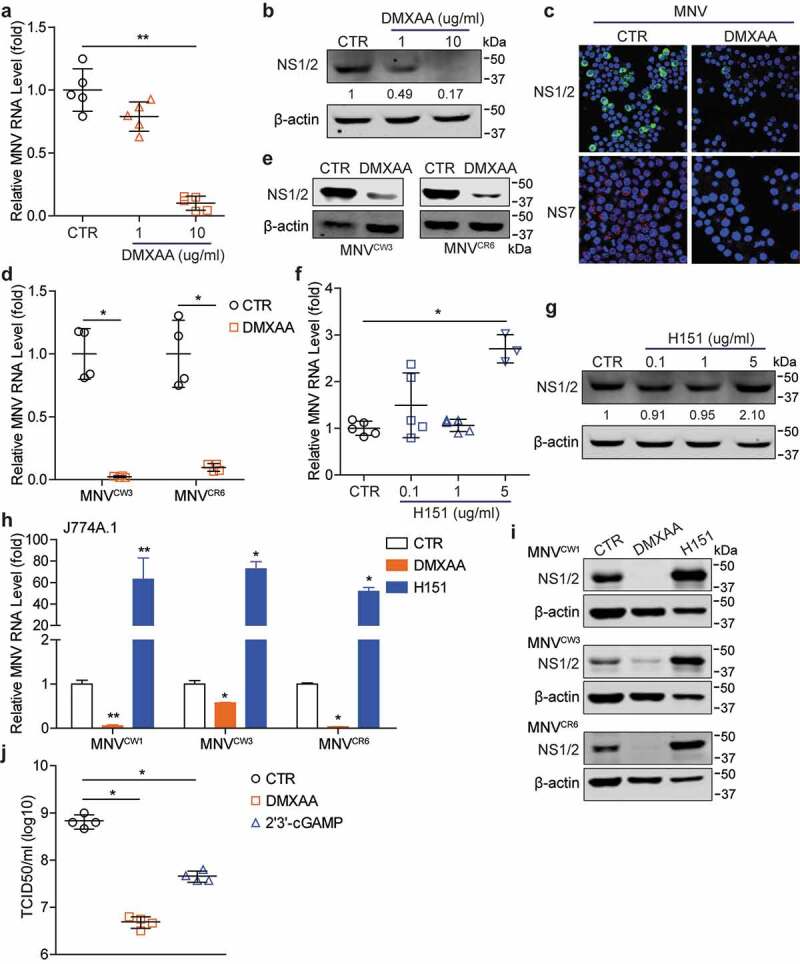
**The effects of DMXAA and H151 on MNV replication in mouse macrophages**. RAW264.7 cells were infected with MNV-1 for 1 h, then untreated or treated with DMXAA with indicated concentrations for 20 h. The viral RNA (a) and NS1/2 protein (b) expression were analyzed by qRT-PCR (n = 5) and western blotting, respectively. (c) RAW264.7 cells were infected with MNV-1 for 1 h, then untreated or treated with DMXAA (10 µg/ml) for 20 h. The viral NS1/2 and NS7 protein expression were analyzed by confocal fluorescence microscopy. RAW264.7 cells were infected with MNV^CW3^ or MNV^CR6^ for 1 h, then untreated or treated with DMXAA (10 µg/ml) for 20 h. The viral RNA (d) and NS1/2 protein (e) expression were analyzed by qRT-PCR (n = 4) and western blotting, respectively. RAW264.7 cells were infected with MNV-1 for 1 h, then untreated or treated with H151 with indicated concentrations for 20 h. The viral RNA (f) and NS1/2 protein (g) expression were analyzed by qRT-PCR (n = 3–5) and western blotting, respectively. J774A.1 cells were infected with indicated MNV strains for 1 h, then untreated or treated with DMXAA (10 µg/ml) or H151 (5 µg/ml) for 20 h. The viral RNA (h) and NS1/2 protein (i) expression were analyzed by qRT-PCR (n = 4–5) and western blotting, respectively. (j) RAW264.7 cells were infected with MNV-1 for 1 h, then untreated or treated with DMXAA (10 µg/ml) or 2ʹ3ʹ-cGAMP (2 µg/ml) for 20 h. The viral titer was analyzed by TCID50 assay (n = 4). Data (a, d, f and h) were normalized to untreated control (set as 1). *P < .05; **P < .01. β-actin was used as a loading control. For immunoblot results (b and g), band intensity of NS1/2 protein in each lane was quantified by Odyssey software, and the quantification results were normalized to β-actin expression (untreated control, set as 1)

Emerging studies have reported the potential involvement of cGAS-STING signaling in restricting RNA virus replication. DMXAA, as an agonist, has been identified to target the STING pathway in a mouse-specific manner,^28^ thus we first tested the effects of DMXAA on MNV replication in mouse macrophages. We found that DMXAA treatment inhibited viral RNA and NS1/2 protein expression (Figure 1(a,b)), showing an 89.93% ± 5.6 (mean ± SD, n = 5, *p* < .01) reduction of viral RNA with 10 µg/ml of DMXAA. The antiviral effect was further confirmed by fluorescent staining showing low viral NS1/2 and NS7 protein expression (Figure 1(c)), and decreased viral titers by TCID50 assay (Figure 1(j)) in DMXAA-stimulated cells. Similar inhibitory effects were found in another two MNV strains, the acutely cleared strain MNV^CW3^ and persistent strain MNV^CR6^ both at viral RNA and NS1/2 protein levels (Figure 1(d,e)). In addition, another STING agonist 2ʹ3ʹ-cGAMP also exerted anti-MNV effects in RAW264.7 cells (Figure 1(j)), and Figure S1a, b.

Based on the antiviral effects of STING agonists, we next tested the role of a STING inhibitor, H151, which can covalently bind to STING and inhibit STING-mediated signaling.^29^ We found that both MNV RNA and NS1/2 protein levels were increased in H151 (5 µg/ml)-treated cells (Figure 1(f,g)). To further determine the involvement of cGAS-STING signaling during MNV replication, another macrophage cell line J774A.1 was used. Similarly, the inhibitory effects on MNV replication were observed in DMXAA- or 2ʹ3ʹ-cGAMP-stimulated cells (Figure 1(h,i), and Figure S1c, d), whereas H151 treatment also facilitated viral replication in this cell line (Figure 1(h,i)). No major cytotoxicity of DMXAA or 2ʹ3ʹ-cGAMP on both RAW264.7 and J774A.1 cells was observed (Figure S1h, and Figure S2e, f). These results suggested potential antiviral effects of cGAS-STING signaling against MNV infection.

### STING agonists mediate MNV inhibition through JAK/STAT pathway

**Figure 2.**
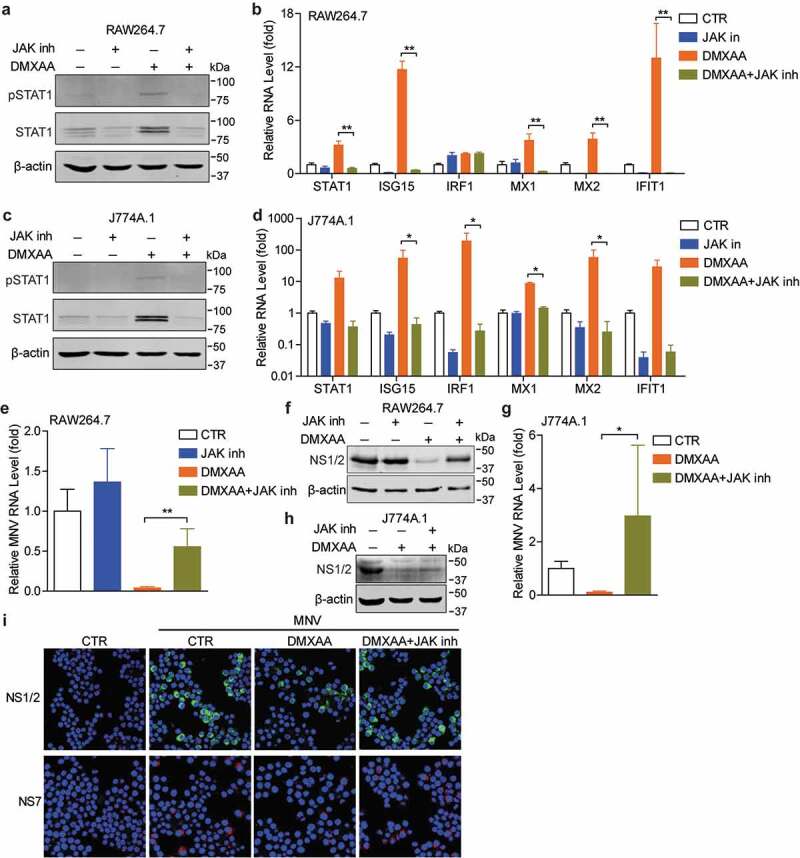
**DMXAA mediates anti-MNV response through JAK/STAT pathway**. (a) Western blotting analysis of STAT1 expression and phosphorylation, and (b) qRT-PCR analysis (n = 5) of mRNA levels of several ISGs in RAW264.7 cells untreated or treated with DMXAA (10 µg/ml) or JAK inhibitor 1 (5 µg/ml) for 20 h. (c) Western blotting analysis of STAT1 expression and phosphorylation, and (d) qRT-PCR analysis (n = 4) of mRNA levels of several ISGs in J774A.1 cells untreated or treated with DMXAA (10 µg/ml) or JAK inhibitor 1 (5 µg/ml) for 20 h. RAW264.7 cells were infected with MNV-1 for 1 h, then untreated or treated with DMXAA (10 µg/ml) or JAK inhibitor 1 (5 µg/ml) for 20 h. The viral RNA (e) and NS1/2 protein (f) expression were analyzed by qRT-PCR (n = 5) and western blotting, respectively. J774A.1 cells were infected with MNV-1 for 1 h, then untreated or treated with DMXAA (10 µg/ml) or JAK inhibitor 1 (5 µg/ml) for 20 h. The viral RNA (g) and NS1/2 protein (h) expression were analyzed by qRT-PCR (n = 4) and western blotting, respectively. (i) RAW264.7 cells were infected with MNV-1 for 1 h, then untreated or treated with DMXAA (10 µg/ml) or JAK inhibitor 1 (5 µg/ml) for 20 h. The viral NS1/2 and NS7 protein expression were analyzed by confocal fluorescence microscopy. Data (b, d, e and g) were normalized to untreated control (set as 1). *P < .05; **P < .01. β-actin was used as a loading control

Since DMXAA treatment of macrophages is associated with the downstream IFN response,^28^ we investigated whether STING agonists-mediated antiviral effects involve ISG response. DMXAA treatment of mouse macrophages increased the expression and phosphorylation of STAT1, and the transcription and protein expression of several antiviral ISGs, including the innate immune sensor MDA5 (Figure S2). Because JAK/STAT cascade is a key component of ISG response, we thus examined whether blocking JAK/STAT pathway could affect DMXAA-mediated ISG response and antiviral activity. We found that treatment with JAK inhibitor attenuated DMXAA-induced STAT1 expression and phosphorylation and downstream ISG transcription in macrophages (Figure 2(a–d)). Moreover, treatment with JAK inhibitor partially attenuated DMXAA-mediated anti-MNV ability in RAW264.7 cells, showing at increased viral RNA and NS1/2 protein levels (Figure 2(e,f)). The attenuated antiviral activity of DMXAA was also seen in JAK inhibitor-treated J774A.1 cells (Figure 2(g,h)). We also observed that inhibition of viral NS1/2 and NS7 protein expression by DMXAA was reversed upon treatment with JAK inhibitor (Figure 2(i)). Similar effects on 2ʹ3ʹ-cGAMP-mediated ISG induction and antiviral activity by JAK inhibitor were also observed (Figure S1).

In addition, we found that conditioned medium from DMXAA-treated cells stimulated IFN response, including increased IFN-β transcription, STAT1 expression and phosphorylation as well as transcription of some antiviral ISGs, whereas these effects were largely attenuated upon treatment with JAK inhibitor in RAW264.7 and J774A.1 cells (Figure S3a-g). The conditioned medium reduced viral RNA and NS1/2 protein levels, whereas the inhibitory effects were also reversed by JAK inhibitor (Figure S3h, i). These results collectively demonstrated that STING agonists mediate anti-MNV effects through JAK/STAT pathway in mouse macrophages.

### Absence of cGAS or STING facilitates MNV replication in mouse macrophages

**Figure 3.**
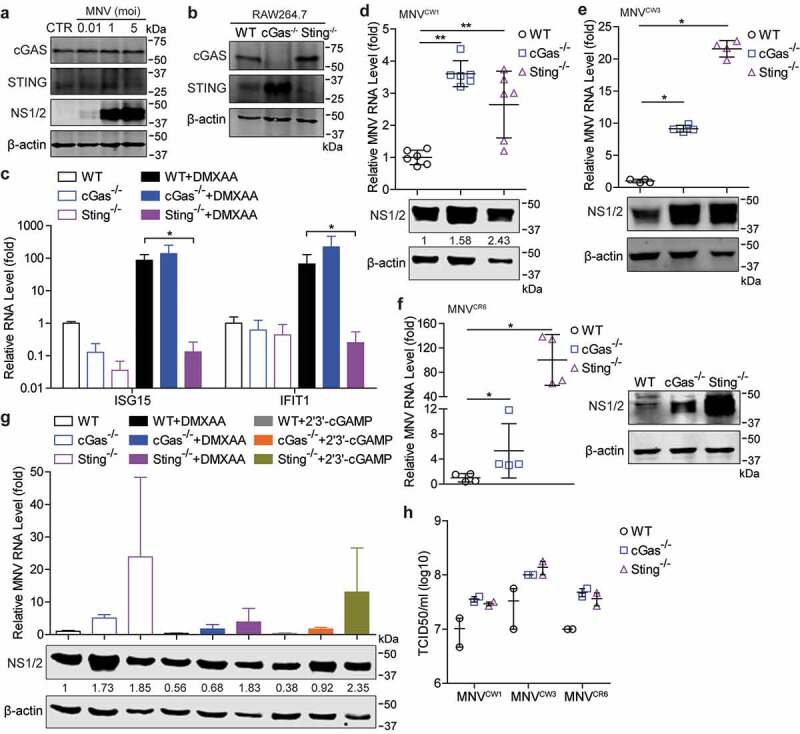
**Absence of cGAS or STING facilitates MNV replication in mouse macrophages**. (a) Western blotting analysis of cGAS and STING expression in RAW264.7 cells uninfected or infected with MNV in indicated MOIs. (b) Western blotting analysis of cGAS and STING expression in cGas^-/-^ and Sting^-/-^ RAW264.7 cells. (c) qRT-PCR (n = 4) analysis of mRNA levels of ISG15 and IFIT1 in the wild-type (WT), cGas^-/-^ and Sting^-/-^ RAW264.7 cells untreated or treated with DMXAA (10 µg/ml) for 20 h. RAW264.7 WT, cGas^-/-^ and Sting^-/-^ cells were infected with MNV-1 for 20 h. (d) The viral RNA and NS1/2 protein expression were analyzed by qRT-PCR (n = 6) and western blotting, respectively. qRT-PCR analysis of viral RNA and western blotting analysis of viral NS1/2 protein expression in WT, cGas^-/-^ and Sting^-/-^ RAW264.7 cells that infected with MNV^CW3^ (e) and MNV^CR6^ (f) for 20 h, respectively. (g) RAW264.7 WT, cGas^-/-^ and Sting^-/-^ cells were infected with MNV-1 for 1 h, then untreated or treated with DMXAA (10 µg/ml) or 2ʹ3ʹ-cGAMP (2 µg/ml) for 20 h. The viral RNA and NS1/2 protein expression were analyzed by qRT-PCR (n = 4) and western blotting, respectively. (h) Viral titers of indicated MNV strains in RAW264.7 WT, cGas^-/-^ and Sting^-/-^ cells were examined by TCID50 assay (n = 2), data are presented as the mean ± SEM. Data were normalized to the WT control (set as 1). *P < .05; **P < .01. β-actin was used as a loading control. For immunoblot results (d and g), band intensity of NS1/2 protein in each lane was quantified by Odyssey software, and the quantification results were normalized to β-actin expression (WT control, set as 1)

Given the potential antiviral effects of STING agonists on MNV replication, we examined whether MNV infection could induce cGAS or STING expression. We found that expression of cGAS and STING appeared not to be changed in response to MNV infection in RAW264.7 cells (Figure 3(a)). To more specifically dissect the role of cGAS-STING signaling on MNV replication, we utilized the cGas or Sting-deficient RAW264.7 cells (Figure 3(b)). We found DMXAA-induced transcription of ISG15 and interferon-induced protein with tetratricopeptide repeats 1 (IFIT1) was largely abrogated in Sting^-/-^ but not cGas^-/-^ cells (Figure 3(c)), further confirming the specificity of DMXAA as a STING ligand. Compared with WT cells, cGas^-/-^ and Sting^-/-^ cells are more permissive in supporting MNV replication evidenced by increased viral RNA and NS1/2 protein expression levels (Figure 3(d)). Similar results were observed when inoculated with the MNV^CW3^ and MNV^CR6^ strains (Figure 3(e,f)). Even with stimulation of DMXAA or 2ʹ3ʹ-cGAMP, the MNV replication levels still increased in cGas^-/-^ and Sting^-/-^ cells compared with WT cells (Figure 3(g)). In addition, we found that the viral titers of the same inoculum on cGas^-/-^ and Sting^-/-^ cells were moderately higher than that on the WT cells (Figure 3(h)), indicating more infectious viruses produced by the deficient cells. These results revealed that cGAS and STING are both necessary for inhibition of MNV replication in mouse macrophages.

### cGAS and STING are necessary for antiviral ISG induction upon MNV infection

**Figure 4.**
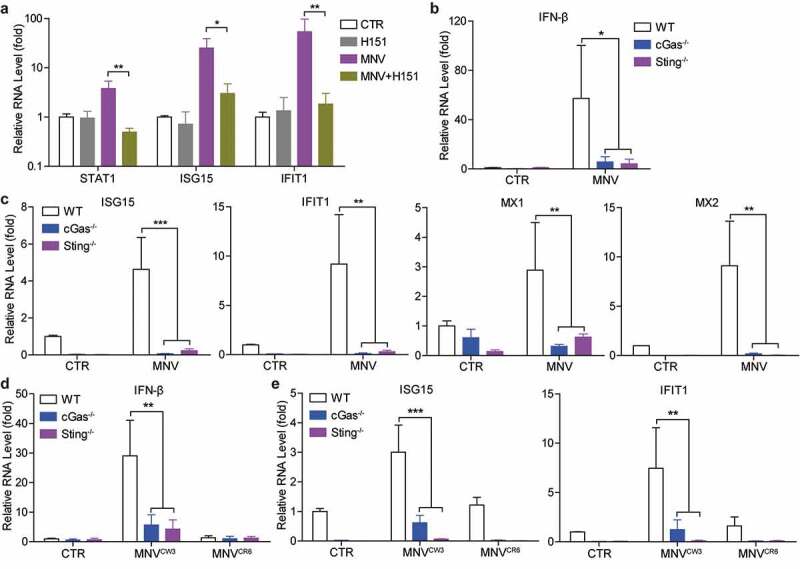
**cGAS and STING are necessary for MNV infection-induced IFN response**. (a) RAW264.7 cells were uninfected or infected with MNV-1 for 1 h, then untreated or treated with H151 (2 µg/ml) for 20 h. The mRNA levels of STAT1, ISG15 and IFIT1 were analyzed by qRT-PCR (n = 5). RAW264.7 WT, cGas^-/-^ and Sting^-/-^ cells were uninfected or infected with MNV-1 for 20 h. The mRNA levels of (b) IFN-β, and (c) ISG15, IFIT1, MX1 and MX2 were analyzed by qRT-PCR (n = 4), respectively. RAW264.7 WT, cGas^-/-^ and Sting^-/-^ cells were uninfected or infected with MNV^CW3^ or MNV^CR6^ for 20 h. The mRNA levels of (d) IFN-β, and (e) ISG15 and IFIT1 were analyzed by qRT-PCR (n = 4), respectively. Data were normalized to the uninfected WT control (set as 1). *P < .05; **P < .01; ***P < .001

IFN-induced ISGs are ultimate antiviral effectors against many viral infections. MNV infection has been shown to induce ISG expression in mouse macrophages and dendritic cells.^24,30^ Thus, we investigated whether cGAS or STING could regulate MNV-induced ISG expression. The results showed that transcription of several ISGs including STAT1, ISG15 and IFIT1 induced by MNV infection were significantly inhibited by H151 treatment in RAW264.7 cells (Figure 4(a)). Consistently, induction of IFN-β and several antiviral ISGs (including ISG15, IFIT1, MX1 and MX2) by MNV infection were dramatically decreased in both cGas^-/-^ and Sting^-/-^ cells, compared to WT cells (Figure 4(b,c)). Notably, basal mRNA levels of tested antiviral ISGs were reduced in uninfected cGas^-/-^ and Sting^-/-^ RAW264.7 cells (Figure 4(c)), which might explain the increased viral replication in these deficient cells. Similar results were found when inoculated with the MNV^CW3^ and MNV^CR6^ strains (Figure 4(d,e)). These data indicated that cGAS and STING are essentially required for MNV-induced antiviral IFN response in mouse macrophages.

### Reconstitution of cGAS or STING expression in the deficient cells restores the antiviral state

**Figure 5.**
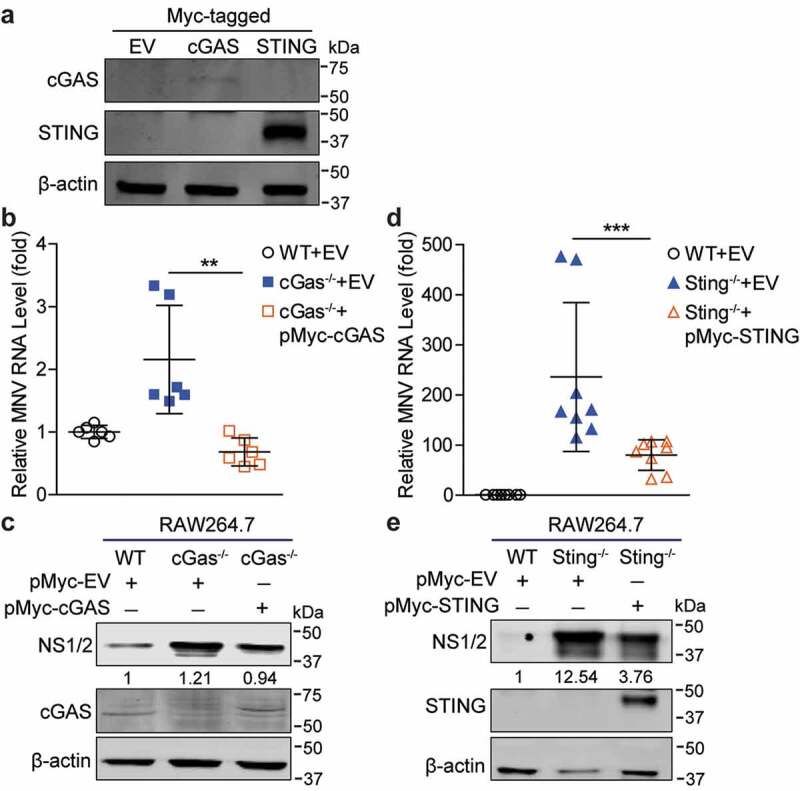
**Reconstitution of cGAS and STING in the deficient cells restores the antiviral state**. (a) HEK293T cells were transfected with pMyc-cGAS (1 µg), pMyc-STING (1 µg) or empty vectors (1 µg) for 24 h. The expression of transfected vectors was detected by western blotting. RAW264.7 WT or cGas^-/-^ cells were transfected with pMyc-cGAS (1 µg) or empty vectors (1 µg) for 24 h, then infected with MNV for 20 h. The viral RNA (b) and NS1/2 protein (c) expression were analyzed by qRT-PCR (n = 6) and western blotting, respectively. RAW264.7 WT or Sting^-/-^ cells were transfected with pMyc-STING (1 µg) or empty vectors (1 µg) for 24 h, then infected with MNV for 20 h. The viral RNA (d) and NS1/2 protein (e) expression were analyzed by qRT-PCR (n = 8) and western blotting, respectively. Data were normalized to the WT control (set as 1). **P < .01; ***P < .001. β-actin was used as a loading control. For immunoblot results (c and e), band intensity of NS1/2 protein in each lane was quantified by Odyssey software, and the quantification results were normalized to β-actin expression in WT cells (set as 1)

Furthermore, we investigated whether reconstitution of cGAS or STING in cGas^-/-^ and Sting^-/-^ cells could restore the anti-MNV effects. We constructed and verified the expression of Myc-tagged plasmids expressing cGAS or STING in HEK293T cells, respectively (Figure 5(a)). Transient expression of cGAS in cGas-deficient cells restored the antiviral state of the cells, evidenced by decreased viral RNA with a 68.34% ± 10.4 (mean ± SD, n = 6, *p* < .01) reduction and NS1/2 protein expression compared with cGas^-/-^ cells transfected with the empty vectors (Figure 5(b,c)). Similarly, reconstitution of STING expression in Sting-deficient cells also restored the antiviral state of these cells, shown by decreased viral RNA with a 66.01% ± 12.9 (mean ± SD, n = 8, *p* < .001) reduction and NS1/2 protein expression (Figure 5(d,e)). These data further confirmed the importance of cGAS and STING in restricting MNV replication in mouse macrophages.

### Overexpression of cGAS and STING restricts MNV replication

**Figure 6.**
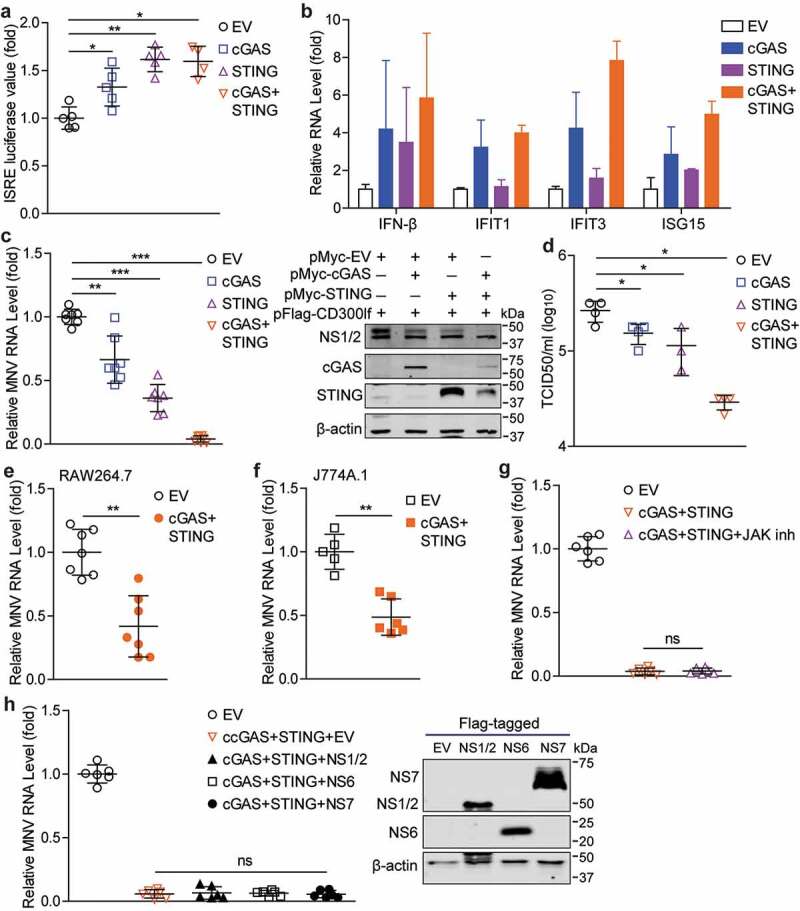
**Overexpression of cGAS and STING restricts MNV replication**. (a) Analysis of ISRE related firefly luciferase activity in Huh7-ISRE-Luc cells transfected with pMyc-cGAS (1 µg), pMyc-STING (1 µg) or empty vectors (1 µg) for 48 h (n = 4–5). (b) HEK293T cells were transfected with pMyc-cGAS (1 µg), pMyc-STING (1 µg) or empty vectors (1 µg) for 48 h. The mRNA levels of IFN-β, IFIT1, IFIT3 and ISG15 were analyzed by qRT-PCR (n = 2–5). HEK293T cells were transfected with pFlag-CD300lf (0.5 µg) and pMyc-cGAS (1 µg), pMyc-STING (1 µg) or empty vectors (1 µg) for 24 h, then infected with MNV-1 for 20 h. (c) Viral RNA, NS1/2 protein and expression of transfected vectors, and (d) viral titers were analyzed by qRT-PCR (n = 7), western blotting and TCID50 assay (n = 3–4), respectively. (e) RAW264.7 (n = 7) and (f) J774A.1 (n = 5–6) cells were transfected with pMyc-cGAS (1 µg), pMyc-STING (1 µg) or empty vectors (1 µg) for 24 h, then infected with MNV-1 for 20 h. The viral RNA levels were analyzed by qRT-PCR. (g) HEK293T cells were transfected with pFlag-CD300lf (0.5 µg), pMyc-cGAS (1 µg), and pMyc-STING (1 µg) or empty vectors (1 µg) for 24 h, then infected with MNV-1 for 1 h, and refreshed medium containing JAK inhibitor 1 (5 µg/ml) for 20 h. The viral RNA levels were analyzed by qRT-PCR (n = 6). (h) Expression of Flag-tagged viral NS1/2, NS6 and NS7 vectors were analyzed by western blotting in HEK293T cells transfected with indicated constructs (1 µg/each). HEK293T cells were transfected with pFlag-CD300lf (0.5 µg), and pMyc-cGAS (1 µg), pMyc-STING (1 µg), pFlag-NS1/2 (1 µg), pFlag-NS6 (1 µg), pFlag-NS7 (1 µg) or empty vectors (were used to maintain a constant amount of plasmid DNA per transfection) for 24 h, then infected with MNV-1 for 20 h. The viral RNA levels were analyzed by qRT-PCR (n = 6). Data were normalized to the EV control (set as 1). *P < .05; **P < .01; ***P < .001. β-actin was used as a loading control

Since human cGAS and STING overexpression can activate antiviral immune response, thus we next evaluated whether overexpression of mouse cGAS and STING could also induce antiviral immune response. By using a transcriptional reporter system that mimics IFN response with a reporter luciferase gene driving by multiple ISREs, we found that expression of cGAS or STING alone, or their combination could increase ISRE-related luciferase activity (Figure 6(a)), and induce a moderate increase in the expression of IFN-β and several antiviral ISGs in HEK293T cells (Figure 6(b)). Because ectopic expression of the MNV receptor (mouse CD300lf) confers human cells to MNV infection,^26,31^ we further characterized whether cGAS and STING overexpression could exert antiviral activity in HEK293T cells. As shown in Figure 6(c,d), overexpression of cGAS or STING significantly inhibited MNV replication, showing at the decreased viral RNA and NS1/2 protein expression as well as virus production in the supernatant. This inhibitory effect is much stronger in the cells that overexpressing both cGAS and STING. In addition, cGAS and STING overexpression also reduced viral RNA levels in mouse macrophages (Figure 6(e,f)).

Overexpression of cGAS and STING had minimal effects on the induction of tested ISGs in RAW264.7 cells (Figure S4), and treatment of JAK inhibitor did not affect cGAS-STING mediated antiviral effects in HEK293T cells (Figure 6(g)), suggesting that cGAS-STING mediated anti-MNV activity is dispensable of ISG induction. Because viral proteins can be the targets of cGAS or STING,^12,17^ we thus further investigated whether the MNV proteins (NS1/2, NS6 and NS7) could affect cGAS-STING mediated antiviral effects. The results showed that cGAS-STING mediated inhibition of MNV replication was not affected by expression of these viral proteins (Figure 6(h)).

### STING but not cGAS interacts with RIG-I, and antagonizes N-terminus of RIG-I mediated antiviral effects

**Figure 7.**
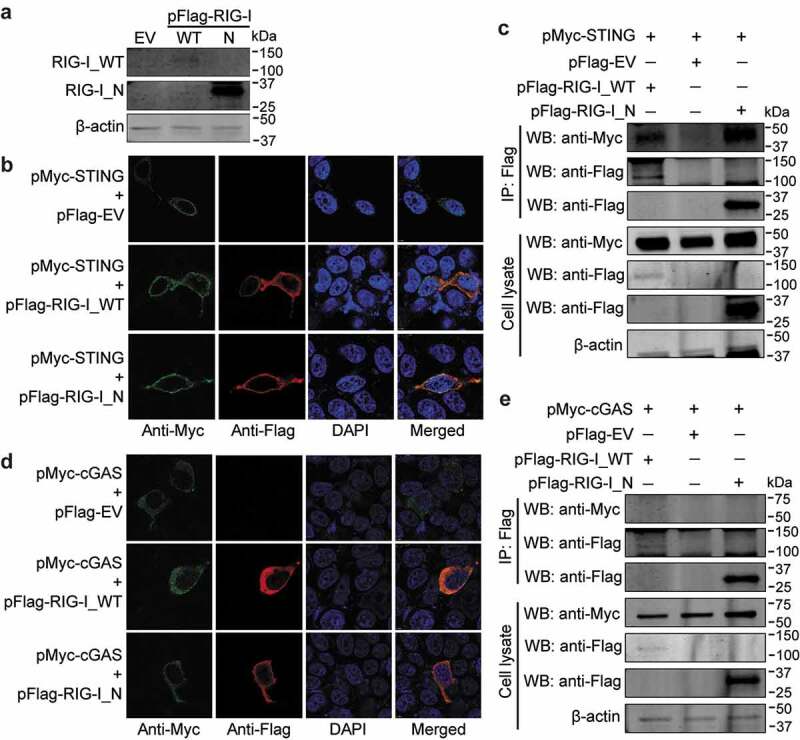
**STING interacts with RIG-I**. (a) HEK293T cells were transfected with pFlag-RIG-I_WT, pFlag-RIG-I_N, or the empty vectors (1 µg/each) for 24 h. Expression of transfected vectors were examined by western blotting. HEK293T cells were transfected with pMyc-STING, and pFlag-RIG-I_WT, pFlag-RIG-I_N or empty vectors (1 µg/each for confocal assay for 24 h; 1.5 µg/each for co-IP assay for 48 h). (b) Expression and co-localization of STING with RIG-I_WT and RIG-I_N were analyzed by confocal assay. (c) Co-IP assay was performed using anti-Flag MAb (1:1000). The precipitated proteins were analyzed by western blotting using anti-Flag and anti-Myc antibodies. HEK293T cells were transfected with pMyc-cGAS, and pFlag-RIG-I_WT, pFlag-RIG-I_N or empty vectors (1 µg/each for confocal assay for 24 h; 1.5 µg/each for co-IP assay for 48 h). (d) Expression and co-localization of cGAS with RIG-I_WT and RIG-I_N was analyzed by confocal assay. (e) Co-IP assay was performed using anti-Flag MAb (1:1000). The precipitated proteins were analyzed by western blotting using anti-Flag and anti-Myc antibodies. β-actin was used as a loading control

Human STING has been reported to interact with RIG-I, a key component of RNA sensing pathway,^11,32^ and this prompted us to investigate whether this occurs in mouse cells. We constructed Flag-tagged vectors expressing WT (wild-type; 2–926 amino acids) and N-terminus (2–235 amino acids) of mouse RIG-I (RIG-I_N), and verified their expression in HEK293T cells (Figure 7(a)). Confocal microscopy revealed that STING could co-localize with either RIG-I or its N-terminus in the cytoplasm (Figure 7(b)), and co-immunoprecipitation assay showed that STING could interact with RIG-I and RIG-I_N (Figure 7(c)). In addition, we also examined the co-localization of cGAS with RIG-I. Although cGAS appeared to co-localize with RIG-I, it was not precipitated by RIG-I or its N-terminus (Figure 7(d,e)). Since MDA5 is a sensor for MNV infection, we further evaluated whether mouse cGAS or STING associates with MDA5. We constructed Flag-tagged vector expressing N-terminus (2–294 amino acids) of mouse MDA5 (MDA5_N), and verified its expression and co-localization with cGAS or STING in HEK293T cells (Figure S5a). Association between MDA5_N with cGAS or STING was not observed by co-immunoprecipitation assay (Figure S5b).

**Figure 8.**
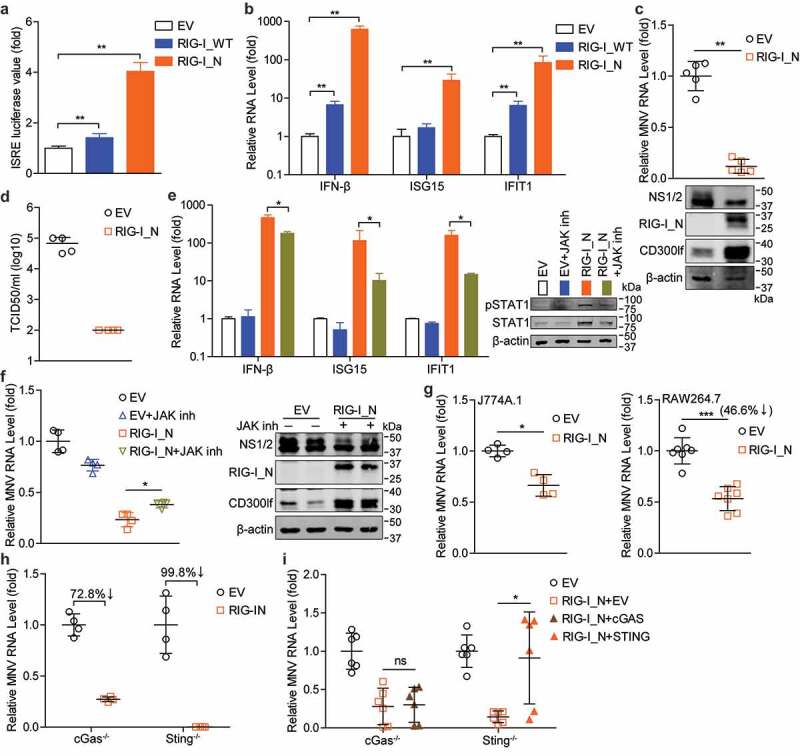
**The N-terminus of RIG-I exerts antiviral effect, but is antagonized by STING expression**. (a) Analysis of ISRE related firefly luciferase activity in Huh7-ISRE-Luc cells transfected with pFlag-RIG-I_WT (1 µg), pFlag-RIG-I_N (1 µg) or empty vectors (1 µg) for 48 h (n = 6). (b) HEK293T cells were transfected with pFlag-RIG-I_WT (1 µg), pFlag-RIG-I_N (1 µg) or empty vectors (1 µg) for 48 h. The mRNA levels of IFN-β, ISG15 and IFIT1 were analyzed by qRT-PCR (n = 5). (c) HEK293T cells were transfected with pFlag-CD300lf (0.5 µg), pFlag-RIG-I_N (1 µg) or empty vectors (1 µg) for 24 h, then infected with MNV for 20 h. The viral RNA, NS1/2 protein and expression of transfected vectors were analyzed by qRT-PCR (n = 5) and western blotting, respectively. (d) HEK293T cells were transfected with pFlag-CD300lf (0.5 µg), pFlag-RIG-I_N (1 µg) or empty vectors (1 µg) for 24 h, then infected with MNV for 20 h. The titers of produced viruses in the supernatant were analyzed by TCID50 assay (n = 4). (e) HEK293T cells were transfected with pFlag-RIG-I_N (1 µg) or empty vectors (1 µg) for 24 h, then untreated or treated with JAK inhibitor 1 (5 µg/ml) for 20 h. The mRNA levels of IFN-β, ISG15 and IFIT1, and STAT1 expression and phosphorylation were analyzed by qRT-PCR (n = 4) and western blotting, respectively. (f) HEK293T cells were transfected with pFlag-CD300lf (0.5 µg), pFlag-RIG-I_N (1 µg) or empty vectors (1 µg) for 24 h, then infected with MNV for 1 h, refreshed medium with or without JAK inhibitor 1 (5 µg/ml) for 20 h. The viral RNA, NS1/2 protein and expression of transfected vectors were analyzed by qRT-PCR (n = 4) and western blotting, respectively. (g) J774A.1 and RAW264.7 cells were transfected with pFlag-RIG-I_N (1 µg) or empty vectors (1 µg) for 24 h, then infected with MNV for 20 h. The viral RNA levels were analyzed in J774A.1 (n = 4) and RAW264.7 (n = 7) cells by qRT-PCR. (h) RAW264.7 cGas^-/-^ or Sting^-/-^ cells were transfected with pFlag-RIG-I_N (1 µg) or empty vectors (1 µg) for 24 h, then infected with MNV for 20 h. The viral RNA levels were analyzed by qRT-PCR (n = 4). (i) RAW264.7 cGas^-/-^ or Sting^-/-^ cells were transfected with pFlag-RIG-I_N (1 µg), pMyc-cGAS (1 µg), pMyc-STING (1 µg), or empty vectors (1 µg) for 24 h, then infected with MNV for 20 h. The viral RNA levels in cGas^-/-^ (n = 6) or Sting^-/-^ (n = 6) RAW264.7 cells were analyzed by qRT-PCR. Data were normalized to the EV control (set as 1). *P < .05; **P < .01; ***P < .001; ns, not significant. β-actin was used as a loading control

Our previous work has revealed that overexpression of human RIG-I restricts norovirus replication partially via ISG induction.^25,26^ Here, we characterized the effects of mouse RIG-I on MNV replication. By using the ISRE luciferase model, we found both the full-length RIG-I and its N-terminus increased ISRE-related luciferase activity (Figure 8(a)). In HEK293T cells, mRNA levels of IFN-β, ISG15 and IFIT1 were increased by RIG-I transfection (Figure 8(b)). It has been reported that the CARD domain in the N-terminus of RIG-I is responsible for signal transduction and activation of IRF-3 and NF-κB, as well as subsequent IFN response.^5^ We found that compared with the full-length RIG-I, its N-terminus induced much stronger levels of ISRE-related luciferase activity and ISG transcription (Figure 8(a,b)). Thus, we further tested the antiviral effects of RIG-I_N, and found that RIG-I_N effectively inhibited viral RNA with a 88.2% ± 6.8 (mean ± SD, n = 5, *p* < .01) reduction and NS1/2 protein expression (Figure 8(c)), as well as production of infectious viruses in the supernatant (Figure 8(d)). RIG-I signaling stimulates downstream ISG expression mainly through JAK/STAT pathway.^33^ We found that JAK inhibitor treatment substantially reduced RIG-I_N induced STAT1 expression and phosphorylation, as well as transcription of IFN-β, ISG15 and IFIT1 (Figure 8(e)). Moreover, RIG-I_N mediated inhibition of MNV replication was partially reversed by JAK inhibitor treatment in HEK293T cells (Figure 8(f)). The inhibitory effects of RIG-I_N against MNV replication were also observed in J774A.1 and RAW264.7 cells, respectively (Figure 8(g)).

As aforementioned, RIG-I_N can interact with STING (Figure 7(c)). We further examined whether STING could regulate RIG-I_N mediated antiviral response. Compared with the WT cells (showing an average 46.6% reduction of viral RNA; Figure 8(g) right panel), the antiviral activity of RIG-I_N was much stronger in cGas^-/-^ or Sting^-/-^ RAW264.7 cells, showing average 72.8% and 99.8% reduction of viral RNA, respectively (Figure 8(h)). Conversely, we found that STING reconstitution in Sting^-/-^ cells significantly attenuated the anti-MNV activity of RIG-I_N, whereas this effect was not seen by cGAS reconstitution in cGas^-/-^ cells (Figure 8(i)). These results revealed that RIG-I_N exerts anti-MNV effect partially through JAK/STAT pathway, but this inhibitory effect is antagonized by STING expression in mouse macrophages.

## Discussion

IFN-mediated innate immune response provides the first line of host defense against viral infections. Within host cells, cyclic dinucleotides (CDNs) can be sensed by STING and stimulate IFN immune response. This antiviral mechanism has been widely described for defending DNA viruses, but emerging evidence indicates this may also function on some RNA viruses.^18,34,35^ Here we demonstrated that two STING agonists, DMXAA and 2ʹ3ʹ-cGAMP, robustly inhibited MNV replication, whereas the STING inhibitor H151 promoted viral replication in mouse macrophages. Experimentally delivery of CDNs into host cells has been shown to activate IFN response and inhibit viral replication,^18^ and DMXAA treatment in macrophages has been reported to activate IFN activation and ISG expression.^28,36^ In this study, both DMXAA and 2ʹ3ʹ-cGAMP can stimulate IFN response and expression of antiviral ISGs in mouse macrophages. Blocking JAK/STAT pathway by JAK inhibitor significantly attenuates DMXAA and 2ʹ3ʹ-cGAMP induced antiviral actions, revealing the importance of antiviral ISG induction for their anti-MNV effects.

Studies have reported that infection of some RNA viruses (such as VSV, SeV, IAV and SINV) do not change STING electrophoretic mobility and stability, indicating that RNA virus infection does not induce hallmarks of STING activation that are associated with IFN response.^19^ Here we also did not observe significant changes in cGAS or STING expression in response to MNV infection. Although a recent study has revealed an inverse effect that STING facilitates human rhinoviruses replication,^37^ most studies have demonstrated that compared with WT cells, the cells lacking cGAS or STING facilitate replication of some RNA viruses such as VSV and WNV.^19,20^ Consistently, our results showed that ablation of cGAS or STING enhances MNV replication, whereas reconstitution of cGAS or STING expression in the deficient cells partially restores the antiviral state. In addition, cGAS is also present in nucleus and nuclear-localized cGAS can enhance antiviral immune response in a non-canonical manner.^22^ Whether mouse cGAS can sense MNV infection in the cytoplasm needs to be further studied. We also revealed that more infectious viruses are produced in cGas- and Sting-deficient cells under same inoculum of MNV strains, which is consistent with previous reports demonstrating that the permissiveness of cells lacking STING was increased in response to VSV infection.^19^ Loss of cGAS or STING has been reported to decrease the basal expression of some antiviral ISGs in uninfected cells,^19,20^ and similar results were also observed in this study. Some studies showed that loss of cGAS or STING impairs RNA virus-induced IFN response, whereas others reported normal level of IFN activation in response to some RNA viruses in the absence of cGAS or STING.^11,19–22^ In this study, compared with the WT cells, MNV infection-induced transcription of some ISGs were reduced in cGas- and Sting-deficient RAW264.7 cells, showing a regulatory role of cGAS and STING on MNV triggered innate immune response. STING has been reported to possess dual functions in host defense by regulating protein synthesis or IFN response to prevent viral infection,^19^ and thus whether this regulation is directly attributed to the loss of cGAS and STING or indirectly through the altered host immune defense status remains to be investigated. We speculate this could also be virus and/or host cell type specific.

MNV can replicate in murine macrophages and dendritic cells, but not in epithelial cells or human cells because of a restriction at viral entry.^31,38,39^ Discovery of the cell-surface expressed MNV receptor (CD300lf) enables MNV infection in human cells (like HeLa and HEK293T cells) and facilitates identification of antiviral cellular factors.^9,31,40^ Overexpression of mouse cGAS and STING moderately upregulates ISG expression, but potently restricts MNV replication in HEK293T cells ectopically expressing viral receptor. However, this antiviral effect is not influenced by JAK inhibitor treatment, suggesting that ISG induction is dispensable for cGAS and STING mediated antiviral effects in human cells, and presenting a distinct antiviral effect compared with STING agonists in the presence of JAK inhibitor. This might be attributed to the difference in cell types: immune vs epithelial cells. Emerging evidence indicates that some RNA viruses have evolved strategies to antagonize the antiviral activity of cGAS and STING. For instance, the nonstructural protein NS4B of yellow fever virus (YFV) interacts STING and inhibits STING-mediated IFN-β promoter-driven luciferase activity.^12^ The DENV NS2B3 protease complex specifically cleaves human STING protein but not mouse STING, and attenuates STING-mediated IFN response,^41^ while the NS2B of DENV degrades cGAS in an autophagy-lysosome-dependent pathway and inhibits IFN production in the infected cells.^17^ Several MNV nonstructural proteins are associated with immune response, including NS1/2 that mediates resistance to IFN-λ modulated clearance of persistent viral infection,^42^ and NS7 as the viral polymerase modulating innate immune response.^26,43^ Here we revealed that these two viral proteins along with NS6 did not exert clear effects on cGAS and STING mediated inhibitory activity, suggesting that these viral proteins are likely not the target of cGAS and STING. Because MNV VF1 has been reported to attenuate RLRs-mediated immune response,^3,44^ and thus whether other viral components including VF1 could influence or are the targets of cGAS and STING mediated antiviral activity needs to be further investigated.

Human STING interacts with RIG-I, and is necessary for RIG-I triggered IFN-β production in murine embryonic fibroblasts.^32^ Besides IFN activation, STING restricts production of both viral and host proteins in a RIG-I/MDA5-dependent manner by initiating global translation inhibition.^19^ Overexpression of human RIG-I or its N-terminus activates IFN response and exerts antiviral activity against HuNV and MNV.^25,26^ Similar actions mediated by N-terminus of mouse RIG-I is also observed in this study, and this antiviral effect partially requires JAK/STAT signaling that triggers ISG induction in HEK293T cells. Unexpectedly, compared with the WT cells, RIG-I_N mediated antiviral effects are augmented in cGas- and Sting-deficient cells, whereas reconstitution of STING but not cGAS in the deficient cells impedes the antiviral effect of RIG-I_N. These results suggest that the interaction between mouse STING and RIG-I_N might affect their antiviral actions. However, we cannot rule out the possible participation of MAVS in our model because human MAVS is known to associate with human STING, although it is not clear whether MAVS directly interacts with STING, or exists as a complex with RIG-I/STING.^32^ Inactivating human RIG-I and MAVS has been reported to not affect HuNV replication,^45,46^ and thus the potential role of basal mouse RIG_I and MAVS expression in response to MNV replication is interesting to be further studied. Theoretically, the RIG-I/MDA5-MAVS axis and cGAS-STING axis are distinct sensing pathways for cytosolic RNA and DNA, respectively. Crosstalk between these two pathways has been documented,^19,32^ but there remain many knowledge gaps. In the context of MNV infection, we encourage future studies to decipher the potential crosstalk between the RIG-I/MDA5-MAVS and cGAS-STING axis.

**Figure 9.**
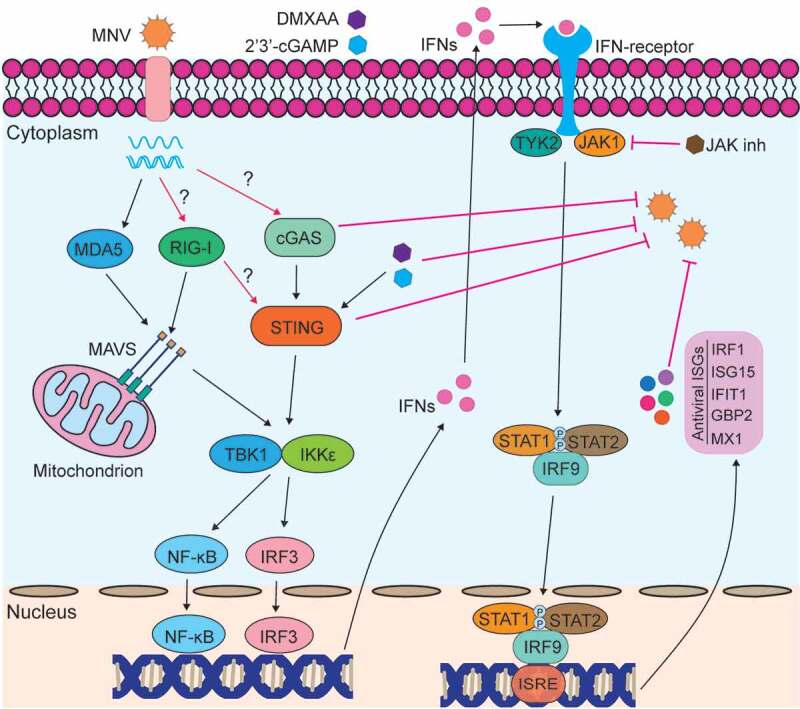
**A proposed model for host immune response upon MNV infection**. Upon infection, MNV is recognized by the pattern recognition receptor MDA5. This activates the downstream cascades, including NF-ĸB and IRF3, inducing production of IFNs. Then, IFNs bind to the receptors and further activate JAK/STAT signaling pathway, leading to transcription of hundreds of ISGs. RIG-I, cGAS and STING can restrict MNV replication in mouse macrophages, but whether they are involved in MNV recognition needs further studies. Also, whether the interaction between RIG-I and STING involving the participation of other host factors needs to be further explored. STING agonists DMXAA and 2ʹ3ʹ-cGAMP inhibit MNV replication through induction of antiviral ISGs, and this effect is partially reversed by JAK inhibitor treatment. The red line with a blunt end indicates the inhibitory effect

In summary, this study reveals that the cGAS-STING signaling essentially contributes to the host defense in response to MNV infection, but these antiviral effects may only be partially through induced antiviral ISGs (Figure 9). Interestingly, mouse STING but not cGAS interacts with RIG-I, and attenuates RIG-I_N mediated inhibition of viral replication in mouse macrophages. Our results implicate a role for mouse cGAS and STING in controlling MNV replication and in regulating innate immune responses, and strengthen the evidence of cGAS-STING signaling in response to RNA virus infection. Nevertheless, future in vivo studies are required to validate our intriguing findings.

## Materials and methods

### Chemicals and antibodies

DMXAA, H151 and 2ʹ3ʹ-cGAMP were purchased from InvivoGen. JAK inhibitor 1 (SC-204021) was purchased from Santa Cruz Biotechnology (Santa Cruz, CA, USA). N-Acetyl-L-cysteine was purchased from Sigma-Aldrich. Rabbit polyclonal antisera to MNV NS1/2 was kindly provided by Prof. Vernon K. Ward (School of Biomedical Sciences, University of Otago, New Zealand).^47^ Rabbit polyclonal antisera to MNV NS7 was kindly provided by Prof. Ian Goodfellow (Department of Pathology, University of Cambridge, UK).^48^ Antibodies against STAT1 (#9172), pSTAT1 (Ser727, #9177), RIG-I (D14G6, #3743), MDA5 (D74E4, #5321), cGAS (D3O8O, #31659), STING (D2P2F, #13647), Myc-tag (71D10, #2278) and Myc-Tag (9B11, #2276) were purchased from Cell Signaling Technology. Rabbit anti-GBP2 (11854-1-AP) and anti-GBP5 (13220-1-AP) antibodies were purchased from Proteintech. Mouse anti-Flag (F1804, Sigma-Aldrich) and anti-β-actin (#sc-47778, Santa Cruz Biotechnology) antibodies were used. Anti-rabbit and anti-mouse IRDye-conjugated secondary antibodies (Li-Cor Bioscience, Lincoln, USA) were used.

### Cell lines

RAW264.7, J774A.1 and human embryonic kidney (HEK293T) cells were cultured in Dulbecco’s modified Eagle’s medium (DMEM; Lonza Verviers, Belgium) supplemented with 10% (vol/vol) heat-inactivated fetal calf serum (FCS; Hyclone, Logan, UT, USA), 100 μg/mL of streptomycin and 100 IU/mL of penicillin. The cGas- and Sting-deficient RAW264.7 cells were kindly provided by Prof. Denise M. Monack (Department of Microbiology and Immunology, Stanford University Stanford School of Medicine).^49^ ISRE (IFN-stimulated response element) activation reporter model (Huh7-ISRE-Luc) was based on Huh7 cells that expressing the firefly luciferase reporter gene driven by a promoter containing multiple ISRE elements (SBI Systems Biosciences, Mountain View, CA). Luciferase activity represents ISRE promoter activation level,^50^ and this cell line was also maintained in DMEM with 10% (vol/vol) heat-inactivated FCS, 100 μg/mL of streptomycin and 100 IU/mL of penicillin.

### Virus strains

Murine norovirus strains MNV-1 (MNV^CW1^), the acutely cleared strain MNV^CW3^ and the persistent strain MNV^CR6^, were produced by consecutively inoculating the virus (kindly provided by Prof. Herbert Virgin, Department of Pathology and Immunology, Washington University School of Medicine) into RAW264.7 cells.^38^ The MNV cultures were purified, aliquoted, and stored at −80℃ for subsequent experiments. The virus stock was quantified by the 50% tissue culture infective dose (TCID50). Without specific statement, the MNV strain used in most part of this study was MNV^CW1^.

### TCID50

TCID50 assay was used to determine the viral titers. Briefly, 10-fold dilutions of MNV were inoculated into RAW264.7 cells grown in 96-well tissue culture plate at 1,000 cells/well. Then the plate was incubated at 37℃ for another 5 days, followed by observing the cytopathic effect (CPE) of each well under a light scope. The TCID50 was calculated by using the Reed-Muench method.

### Plasmid construction and cell transfection

The mouse cGAS and STING genes were amplified from a cDNA sample that isolated from RAW264.7 cells, and cloned into pcDNA3.1/Myc-His vector (kindly provided by Dr. Shuaiyang Zhao, Chinese Academy of Agricultural Sciences, China), respectively. The full-length mouse RIG-I gene and its N-terminus, and the N-terminus of mouse MDA5 were amplified from a cDNA sample that isolated from RAW264.7 cells and cloned into pcDNA3.1/Flag-HA vector (Addgene), respectively. The viral genes NS1/2 and NS6 were amplified from a cDNA sample that isolated from MNV-1 infected RAW264.7 cells, and cloned into pcDNA3.1/Flag-HA vector, respectively. Flag-tagged plasmid expressing MNV NS7 was previously described.^9^ Flag-tagged plasmid expressing the MNV receptor CD300lf was kindly provided by Prof. Herbert Virgin (Department of Pathology and Immunology, Washington University School of Medicine, USA).^31^ The primer sequences used for plasmid construction are shown in Table S1.

The cells were transfected with various plasmids at indicated concentrations using FuGENE HD Transfection Reagent (E2311; Promega) according to the manufacturer’s instructions. Where necessary, appropriate empty vectors were used to maintain a constant amount of plasmid DNA per transfection.

### RNA isolation and qRT-PCR

The Macherey NucleoSpin RNA II Kit (Bioke, Leiden, The Netherlands) was used for total RNA isolation. The cDNA synthesis kit (TaKaRa Bio, Inc., Shiga, Japan) was used to synthesize cDNA from RNA samples, then targeted genes were quantified by SYBR-Green-based (Applied Biosystems) real-time PCR on the StepOnePlus^TM^ System (Thermo Fisher Scientific LifeSciences) according to the manufacturer’s instructions. Human and mouse glyceraldehyde-3-phosphate dehydrogenase (GAPDH) genes were used as reference genes to normalize gene expression. The relative expression of targeted genes were calculated using the 2^−ΔΔCT^ method, and primer sequences used for quantification of targeted genes are shown in Table S2.

### Western blotting

The cells were lysed in Laemmli sample buffer containing 0.1 M DTT and heated 5 min at 95°C, then loaded onto a 10% or 12% sodium dodecyl sulfate polyacrylamide gel electrophoresis (SDS-PAGE) gel. Then proteins were further electrophoretically transferred onto a polyvinylidene difluoride (PVDF) membrane (pore size, 0.45 μM; Invitrogen) for 1.5 or 2 h with an electric current of 250 mA. Then, the membrane was blocked with a mixture of 2.5 mL blocking buffer (Odyssey) and 2.5 mL PBS containing 0.05% Tween 20 for 1 h, followed by overnight incubation with appropriate primary antibodies at 4°C. After washing 3–4 times, the membrane was incubated with appropriate IRDye-conjugated secondary antibodies for 1 h. Then, after washing 3 times, protein bands on the membrane were detected with the Odyssey 3.0 Infrared Imaging System (Li-Cor Biosciences).

### Confocal fluorescence microscopy

RAW264.7 cells (3 × 10^4^ cells/well) were seeding into µ-slide 8-well chamber (Cat. no. 80826; ibidi GmbH) and were rested at 37°C overnight. The cells were either left uninfected or infected with MNV for 1 h at 37°C, and the virus inoculum was removed and cells were washed two times with phosphate-buffered saline (PBS). Then, fresh medium containing DMXAA or JAK inhibitor 1 were added back onto cells for 20 h. For co-localization of mouse RIG-I or MDA5_N with cGAS or STING, HEK293T cells (3 × 10^4^ cells/well) were co-transfected with pMyc-cGAS, pMyc-STING or pFlag-RIG-I_WT, pFlag-RIG-I_N, pFlag-MDA5_N (1 µg/each) into µ-slide 8-well chamber for 24 h. The cells were washed and fixed with 4% paraformaldehyde in PBS, permeablized with 0.2% Triton X-100, blocked with blocking buffer (Odyssey) for 1 h, reacted with the rabbit polyclonal antisera to MNV NS1/2 and NS7, or anti-Flag and anti-Myc antibodies, and stained with 4ʹ,6-diamidino-2-phenylindole (DAPI). Anti-rabbit IgG (H + L), F(ab’)2 Fragment (Alexa Fluor® 488 and 594 conjugate) and Anti-mouse IgG (H + L), F(ab’)2 Fragment (Alexa Fluor® 594 conjugate) antibodies were used as secondary antibodies. Imaging was performed on a Leica SP5 confocal microscopy using a 63x oil objective.

### Co-immunoprecipitation

HEK293T cells (1 × 10^5^ cells/well) were co-transfected with pMyc-cGAS, pMyc-STING or pFlag-RIG-I_WT, pFlag-RIG-I_N, pFlag-MDA5_N (1.5 µg/each) in 12-well tissue culture plate for 48 h. Then the cells were washed twice with cold PBS and lysed with cold NP-40 lysis buffer at 4°C for 30 minutes. Halt™ Protease Inhibitor Cocktail (Thermo Fisher Scientific) was used during lysis according to the manufacturer’s instructions. The cells collected by scraping and lysates were cleared by centrifugation at 12,000 rpm for 10 minutes at 4°C. 10% of the supernatants were taken as input control, and the remaining supernatants were incubated with mouse anti-Flag antibody at 4°C for 2 h, and then incubated with protein A/G plus-agarose (sc-2003; Santa Cruz) overnight at 4°C. The agaroses were centrifuged and washed three times, and the bound proteins were analyzed by western blotting.

### Antiviral assay with MNV

RAW264.7 wild-type (WT), cGas- or Sting-deficient cells, and J774A.1 (3 × 10^4^ cells/well) were uninfected or infected with MNV for 1 h at 37°C, and washed two times with PBS, and then fresh medium or medium containing indicated compounds were added back onto cells for 20 h in 48-well tissue culture plate. The total RNA, supernatant or protein samples were collected for further analysis. To determine the potential antiviral effects of mouse cGAS, STING or RIG-I_N, cells were transfected with pMyc-cGAS, pMyc-STING or pFlag-RIG-I_N with indicated concentrations for 24 h, then infected with MNV for 20 h. For transfection of HEK293T cells, the cells were co-transfected with pFlag-CD300lf, whereas no pFlag-CD300lf was transfected into mouse macrophages. To investigate the role of viral NS1/2, NS6 and NS7 on cGAS and STING mediated antiviral effects, HEK293T cells were co-transfected with pFlag-CD300lf, pMyc-cGAS, pMyc-STING or Flag-tagged vectors expressing indicated viral proteins for 24 h, then the cells were washed and infected with MNV for 20 h. The total RNA, supernatant or protein samples were collected for further analysis.

### Measurement of luciferase activity

Luciferin potassium salt (Sigma-Aldrich, Zwijndrecht, the Netherlands) was added to cells at a final concentration of 0.1 mM for 30 min at 37°C. The luciferase activity was measured with a LumiStar Optima luminescence counter (BMG Lab Tech, Offenburg, Germany).

### MTT assay

RAW264.7 or J774A.1 cells were seeded into 96-well tissue culture plates and treated with compounds for indicated time periods. The cell viability was assessed by adding 10 mM 3-(4,5-dimethyl-2-thiazolyl)-2,5-diphenyl-2 H-tetrazolium bromide (MTT) (Sigma, Zwijndrecht, the Netherlands). After 3 h, the medium was replaced with DMSO (100 μL), and was incubated at 37°C for 30 mins. The absorbance at 490 nm was recorded on the microplate absorbance reader (Bio-Rad, CA, USA).

### Statistical analysis

Data are presented as the mean ± SD without specific statement. Statistical analysis was performed using GraphPad Prism 5.0 (GraphPad Software Inc., La Jolla, CA, USA). Comparisons between two groups were performed with Mann–Whitney test. Comparisons among multiple groups were performed with one-way ANOVA with Newman–Keuls test. Differences were considered significant at a *P* value less than 0.05.

## Supplementary Material

Supplemental Material

## Data Availability

The authors confirm that the data supporting the findings of this study are available within the article and its supplementary materials.
