# Supplementary material for: cGAS-STING effectively restricts murine norovirus infection but antagonizes the antiviral action of N-terminus of RIG-I in mouse macrophages
Source: Gut Microbes. 2021 Aug 4;13(1):1959839. doi: 10.1080/19490976.2021.1959839 (PMC8344765; doi:10.1080/19490976.2021.1959839)
Supplement: Supplemental Material [file KGMI_A_1959839_SM7135.pdf]

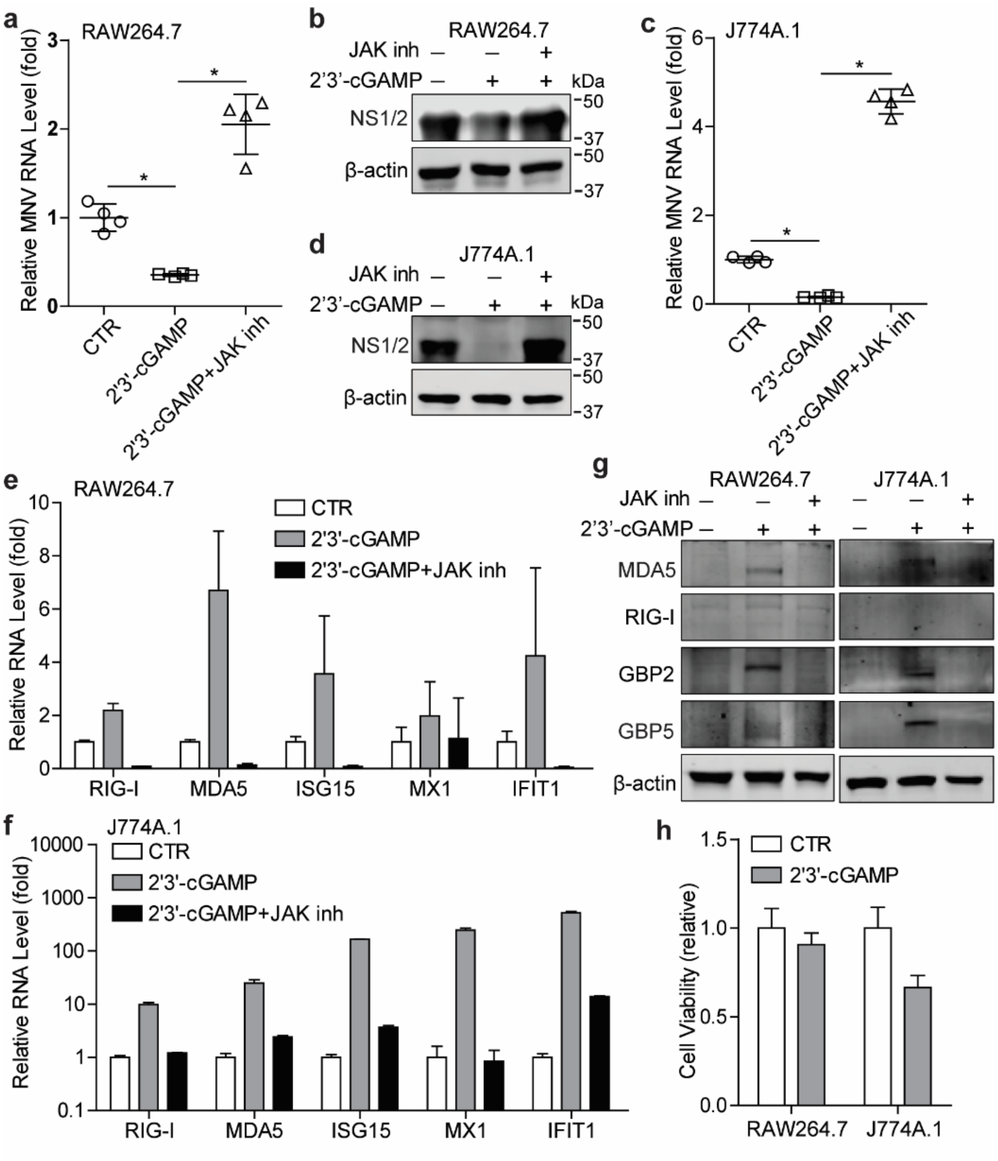

Figure S1. 2'3'-cGAMP restricts MNV replication. RAW264.7 cells were infected with MNV-1 for 1 h, then untreated or treated with 2'3'-cGAMP (2 µg/ml) or JAK inhibitor 1 (5 µg/ml) for 20 h. The viral RNA (a) and NS1/2 protein (b) expression levels were analyzed by qRT-PCR (n = 4) and western blotting, respectively. J774A.1 cells were infected with MNV-1 for 1 h, then untreated or treated with 2'3'-cGAMP (2 µg/ml) or JAK inhibitor 1 (5 µg/ml) for 20 h. The viral RNA (c) and NS1/2 protein (d) expression levels were analyzed by qRT-PCR (n = 4) and western blotting, respectively. qRT-PCR analysis of mRNA levels of RIG-I, MDA5, ISG15, MX1 and IFIT1 in RAW264.7 (e, n = 3-4) and J774A.1 (f, n = 2) cells untreated or treated with 2'3'-cGAMP (2 µg/ml) or JAK inhibitor 1 (5 µg/ml) for 20 h. (g) RAW264.7 or J774A.1 cells

were untreated or treated with 2’3’-cGAMP (2 µg/ml) or JAK inhibitor 1 (5 µg/ml) for 20 h. Expression of MDA5, RIG-I, GBP2 and GBP5 proteins were analyzed by western blotting. (h) RAW264.7 or J774A.1 cells were untreated or treated with 2’3’-cGAMP (2 µg/ml) for 20 h. The cytotoxicity was determined by MTT assay (n = 16). Data were normalized to untreated control (set as 1). *P < 0.05. β-actin was used as a loading control.

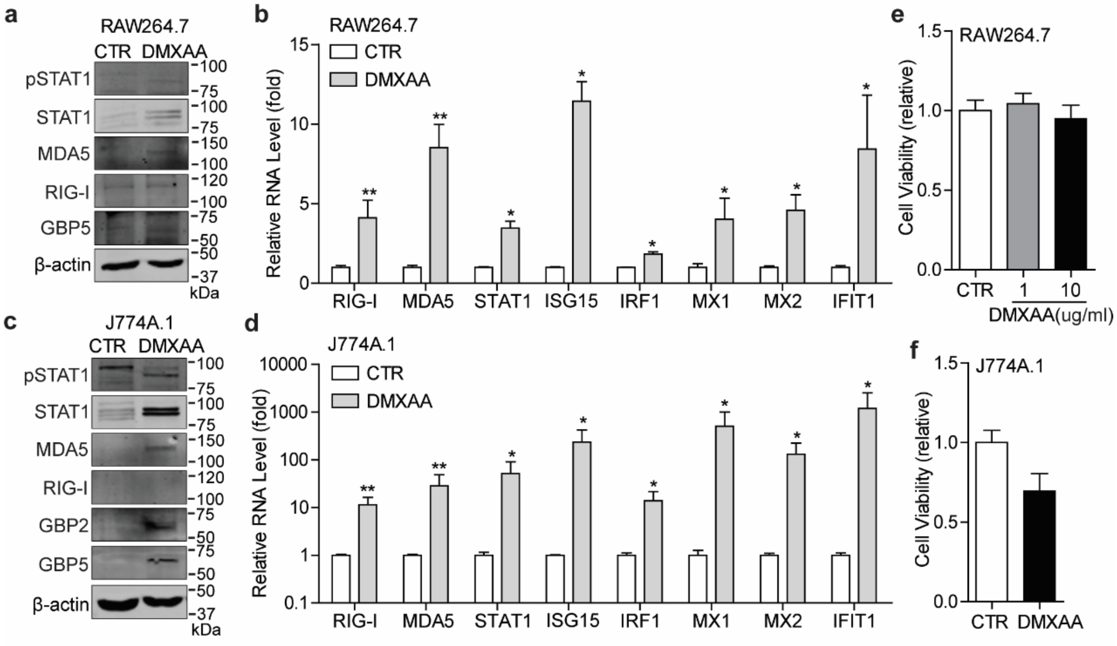

Figure S2. DMXAA treatment induces ISG transcription. RAW264.7 cells were untreated or treated with DMXAA (10 µg/ml) for 20 h. (a) STAT1 expression and phosphorylation, MDA5, RIG-I and GBP5 expression, and (b) mRNA levels of indicated ISGs were analyzed by western blotting and qRT-PCR (n = 4-5), respectively. J774A.1 cells were untreated or treated with DMXAA (10 µg/ml) for 20 h. (c) STAT1 expression and phosphorylation, MDA5, RIG-I, GBP2 and GBP5 expression, and (d) mRNA levels of indicated ISGs were analyzed by western blotting and qRT-PCR (n = 4-5), respectively. (e) RAW264.7 cells were untreated or treated with DMXAA (1 or 10 µg/ml) for 20 h. The cytotoxicity was determined by MTT assay (n = 16). (f) J774A.1 cells were untreated or treated with DMXAA (10 µg/ml) for 20 h. The cytotoxicity was determined by MTT assay (n = 16). Data were normalized to the untreated control (set as 1). *P < 0.05; **P < 0.01. β-actin was used as a loading control.

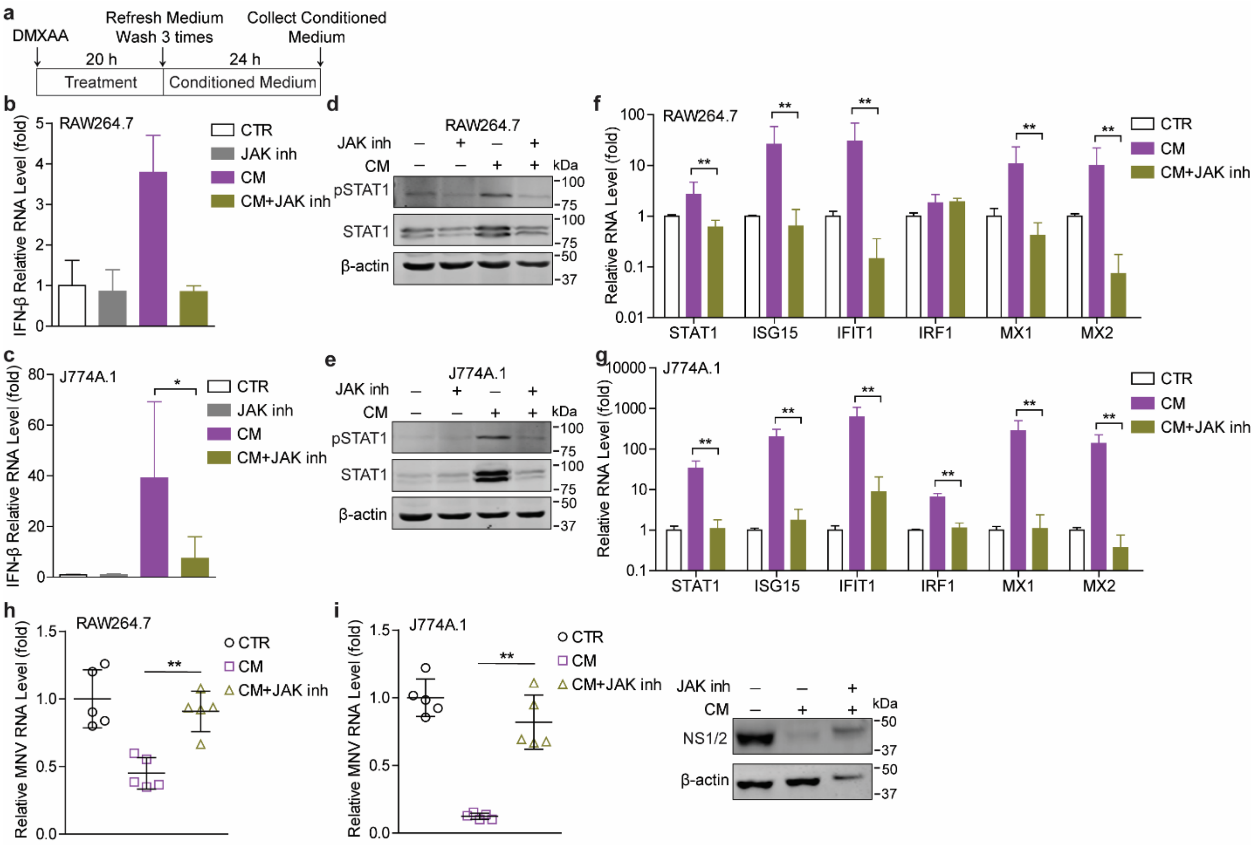

Figure S3. The conditioned medium (CM) from DMXAA-treated cells exerts anti-MNV activity. (a) Production of conditioned medium (supernatant). RAW264.7 or J774A.1 cells were treated with DMXAA (10 µg/ml) for 20 h, then were washed 3 times and medium was refreshed. Cells were further cultured for 24 h and supernatant was collected as conditioned medium. RAW264.7 and J774A.1 cells were untreated or treated with JAK inhibitor 1 (5 µg/ml) or conditioned medium (CM) from DMXAA-treated RAW264.7 or J774A.1 cells for 20 h, respectively. The IFN-β mRNA level in (b) RAW264.7 (n = 2) and (c) J774A.1 (n = 5) cells, and mRNA levels of several ISGs in (f) RAW264.7 (n = 5) and (g) J774A.1 (n = 5) cells were analyzed by qRT-PCR, respectively. Western blotting analysis of STAT1 expression and phosphorylation in (d) RAW264.7 and (e) J774A.1 cells untreated or treated with JAK inhibitor 1 (5 µg/ml) or CM from DMXAA-treated RAW264.7 or J774A.1 cells for 20 h. (h) RAW264.7 cells were infected with MNV-1 for 1 h, then untreated or treated with CM from DMXAA-treated RAW264.7 cells or combinations with JAK inhibitor 1 (5 µg/ml) for 20 h. The viral RNA level was analyzed by qRT-PCR (n = 5). (i) J774A.1 cells were infected with MNV-1 for 1 h, then untreated or treated with CM from DMXAA-treated J774A.1 cells or combinations with

JAK inhibitor 1 (5 μg/ml) for 20 h. The viral RNA and NS1/2 protein expression levels were analyzed by qRT-PCR (n = 5) and western blotting, respectively. Data were normalized to the untreated control (set as 1). *P < 0.05; **P < 0.01. β-actin was used as a loading control.

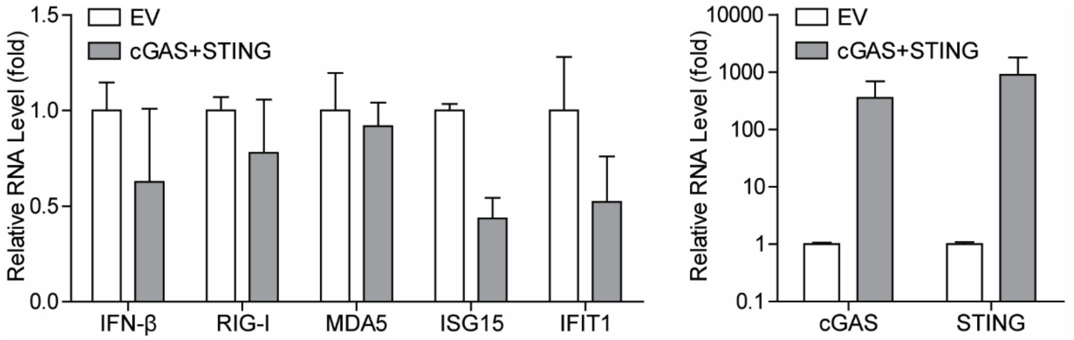

Figure S4. RAW264.7 cells were transfected with pMyc-cGAS and pMyc-STING (1 ug/well) or empty vectors for 48 h. The mRNA levels of indicated genes were analyzed by qRT-PCR (n = 4). Data were normalized to the EV control (set as 1).

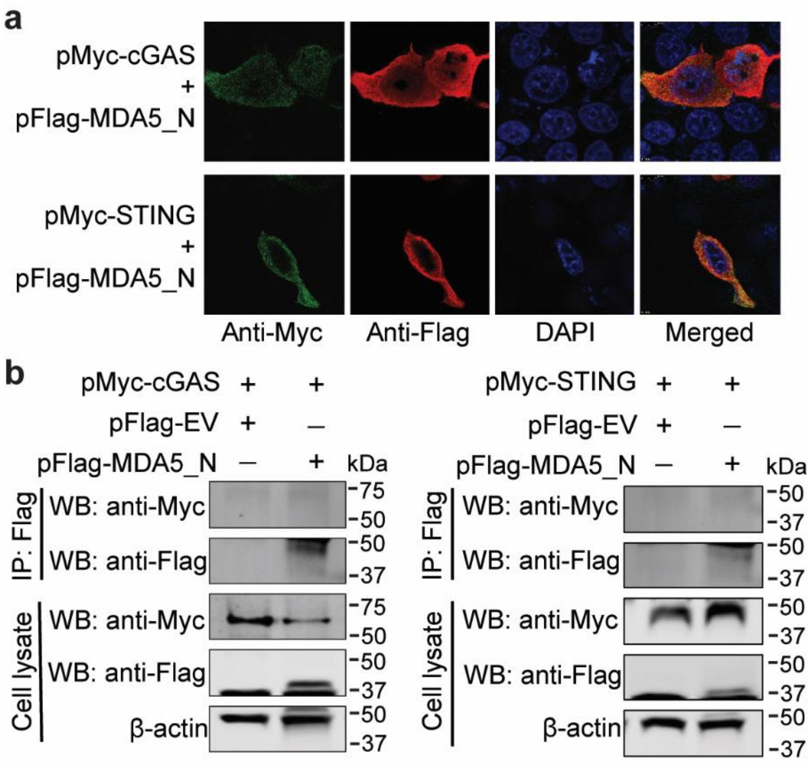

Figure S5. (a) HEK293T cells were transfected with pFlag-MDA5_N and pMyc-cGAS or pMyc-STING (1 µg/each) for 24 h. Expression and co-localization of MDA5_N with cGAS or STING were analyzed by confocal assay. (b) HEK293T cells were transfected with pMyc-cGAS or pMyc-STING and pFlag-MDA5_N or empty vectors (1.5 µg/each) for 48 h. Co-IP assay was performed using anti-Flag MAb (1:1000). The precipitated proteins were analyzed by western blotting using anti-Flag and anti-Myc antibodies. β-actin was used as a loading control.

Table S1. The primers used for plasmid construction

Primer	Sequence (5'to 3')	Usage
pMyc-cGAS-F	CCGCTCGAGATGGAAGATCCGCGTAGA	Amplification of
pMyc-cGAS-R	GGGGTACCAAGCTTGTCAAAAATTGG	mouse cGAS
pMyc-STING-F	CTAGCTAGCATGCCATACTCCAACCTGC	Amplification of
pMyc-STING-R	CCAAGCTTGATGAGGTCAGTGCGGAGTG	mouse STING
pFlag-NS1/2-F	CCGCTCGAGAGGATGGCAACGCCATCTTCTG	Amplification of
pFlag-NS1/2-R	CGGGGTACCTTATTCGGCCTGCCATTCCCCGA	MNV NS1/2
pFlag-NS6-F	CCGCTCGAGGCCCCAGTCTCCATCTGGTCCC	Amplification of
pFlag-NS6-R	CGGGGTACCTTACTGGAACTCCAGAGCCTCAAG	MNV NS6
pFlag-RIG-I_WT-F	TGCTCTAGAACAGCGGAGCAGCGGCAGAATCTG	Amplification of full-
pFlag-RIG-I_WT-R	CGGGGTACCTCATACGGACATTTCTGCAGGATC	length mouse RIG-I
pFlag-RIG-I_N-F	TGCTCTAGAACAGCGGAGCAGCGGCAGAATCTG	Amplification of
pFlag-RIG-I_N-R	CGGGGTACCTCAAGAAGACGCTTCTGAAGG	mouse RIG-I_N
pFlag-MDA5_N-F	CCGCTCGAGTCGATTGTCTGTTCTGCAG	Amplification of
pFlag-MDA5_N-R	CGGGGTACCCTAACTTTCATCTGAATCAC	mouse MDA5_N

Table S2. The primers used for qRT-PCR

Primer	F-Sequence (5'to 3')	R-Sequence (5' to 3')
mGAPDH	TTCCAGTATGACTCCACTCACGG	TGAAGACACCAGTAGACTCCACGAC
mSTAT1	GCCTCTCATTGTCACCGAAGAAC	TGGCTGACGTTGGAGATCACCA
mIRF1	CAGAGGAAAGAGAGAAAGTCC	CACACGGTGACAGTGCTGG
mISG15	TGACGCAGACTGTAGACACG	TGGGGCTTTAGGCCATACTC
mMX1	GGGGAGGAAATAGAGAAAATGAT	GTTTACAAAGGGCTTGCTTGCT
mMX2	CCAGTTCCTCTCAGTCCCAAGATT	TACTGGATGATCAAGGGAACGTGG
mIFIT1	CCATAGCGGAGGTGAATATC	GGCAGGACAATGTGCAAGAA
mIFN-β	AAGAGTTACACTGCCTTTGCCATC	CACTGTCTGCTGGTGGAGTTCATC
mRIG-I	AGCCAAGGATGTCTCCGAGGAA	ACACTGAGCACGCTTTGTGGAC
mMDA5	GTGATGACGAGGCCAGCAGTTG	ATTCATCCGTTTCGTCCAGTTTCA
mcGAS	GGCAGCTACTATGAACATGTG	CTCAGCGGATTTCCTCGTGGAA
mSTING	TATACCTCAGTTGGATGTTTGGC	CTGGAGTCAAGCTCTGAAGGC
hGAPDH	TGTCCCCACCCCCAATGTATC	CTCCGATGCCTGCTTCACTACCTT
hIFN-β	CTTGGATTCCTACAAAGAAGCAGC	TCCTCCTTCTGGAACTGCTGCA
hISG15	CTCTGAGCATCCTGGTGAGGAA	AAGGTCAGCCAGAACAGGTCGT
hIFIT1	GCCTTGCTGAAGTGTGGAGGAA	ATCCAGGCGATAGGCAGAGATC
hIFIT3	CCTGGAATGCTTACGGCAAGCT	GAGCATCTGAGAGTCTGCCCAA
MNV-1	CACGCCACCGATCTGTTCTG	GCGCTGCGCCATCACTC
